# CNS Depressant Activity of Ethanol Extract of *Sterculia guttata* seeds in mice

**DOI:** 10.4103/0250-474X.57297

**Published:** 2009

**Authors:** S. R. Katade, A. V. Misar, A. M. Mujumdar, U. D. Phalgune, R. D. Wakharkar, N. R. Deshpande

**Affiliations:** T. R. Ingle Research Laboratory, Department of Chemistry, Sir Parshurambhau College, Pune-411 030, India; 1 Division of Plant Sciences, Agharkar Research Institute, Pune-411 004, India; 2 Center for Materials Characterization, National Chemical Laboratory, Pune-411 008, India; 3 Organic Chemistry Technology, National Chemical Laboratory, Pune-411 008, India

**Keywords:** Barbitone sodium, CNS depressant activity, pentobarbitone sodium, *Sterculia guttata* Roxb., Swiss albino mice

## Abstract

Shade dried, powdered seeds of *Sterculia guttata* were extracted using a Soxhlet extractor with ethanol. Ethanol was removed under reduced pressure and dried to obtained crude extract. This extract was evaluated for its effect on behavioral changes, exploratory activity and barbiturate-sleeping time, using appropriate standard methods in mice. The extract exhibited dose-dependent CNS depressant activity.

*Sterculia guttata* Roxb. (Sterculiaceae) is an important medicinal tree that is distributed in India in Maharashtra, Assam and Andamaans[1]. The bark fiber is used for cordage and rough fabrics. This plant is reported as a famine food. The seeds are eaten raw or roasted by tribes, especially at the time of scarcity[1–3]. The juice obtained from the bark and *phangali* (*Pogosteman benghalensis*) leaves by crushing in water, is used in folk medicine to cure fever and diarrhoea[3]. A literature survey revealed the presence of malvelic and sterculic (2.1 and 5.8%, respectively), hexadecanoic (palmitic), octadecanoic (stearic), 9,12-octadecedienoic(Z,Z) (linoleic), 9-octadecenoic (Z) (oleic) and 9-hexadecenoic (Z) (palmitoleic) acids from the seeds of *Sterculia guttata*[4].

It was observed and experienced during the field work that tribal mainly Katkaries consume the seeds of *S. guttata*, especially during the scarcity of food. It was observed that after eating a handful of seeds, they felt sleepy. The leaf extract of *Sterculia foetida* of sterculiaceae species has been reported for CNS depressant activity[5]. On the basis of the information obtained from the tribes, the investigation of seeds of *S. guttata* for CNS depressant activity has been explored.

The fruits of *S. guttata* were collected from deciduous forests near Pune, India in bulk quantity. The plant specimen was authenticated by matching with the voucher specimen BSI/WC/Tech/2000/358 available at the Botanical Survey of India, Pune, India. Shade dried, powdered material of *S. guttata* seeds (180.0 g) were extracted using a Soxhlet extractor with ethanol. Ethanol was removed under reduced pressure and temperature to obtain a crude extract (26.49%).

The experiments were carried out on Swiss mice, which were originally obtained from the National Institute of Virology, Pune. They have been bred in the animal house facility at Agharkar Research Institute (ARI), Pune, India, for several generations for the last 19 y. They were housed in polypropylene cages in an air-conditioned area at 25±2° with 10 to 14 h light and dark cycle. They were given Amrut brand balanced animal feed and water *ad libitum.* For each pharmacological study a group of at least six animals was used for individual treatment. Animal experiments were conducted after obtaining the permission of the Institutional Animal Ethics Committee (IAEC) of ARI.

The acute toxicity profile of above extract was performed according to OECD guideline No. 423 using mice. Limit test was performed by using a dose of 2000 mg/kg by oral route. The animals were observed for 14 days for any sign, symptoms and mortality. There was no mortality up to 2000 mg/kg dose. It was observed that there was significant reduction in the activity of animals from ½ h up to 4 h and subsequently they were normal.

Based on these observations further studies were carried out after administration of 500 and 1000 mg/kg dose by oral route 30 min prior to testing the following CNS activities; exploratory activity was evaluated using the hole-board method with the help of a board (40×40 cm) with four equidistance holes (1 cm diameter, 2 cm depth). The mouse was placed at one corner of the board and the animal moved about and dipped its head into the holes indicating exploratory behavior. The number of dips in 7.5 min was recorded according to File and Wardrill[6]. The test was carried out 30 min after treatment with extract to various groups of mice, using chlorpromazine HCl, 1 mg/kg as a standard. There was significant dose-dependent reduction in exploratory activity in mice pre-treated with *Sterculia guttata* extract as compared to control mice; however, it was less as compared to chlorpromazine HCl as shown in Table 1.

**TABLE 1:** EFFECT OF ETHANOL EXTRACT OF SEED OF *STERCULIA GUTTATA* ON EXPLORATORY ACTIVITY

Pre-treatment	No. of head dips mean±SEM (in 7.5 min)
Control	13.83±1.61
Extract 500 mg/kg	10.17±0.68*
Extract 1000 mg/kg	7.00±0.73*
Chlorpromazine HCl 1 mg/kg	6.77±0.94*

n=6, All values for head dips are expressed as mean±SEM,

* Significant as compared to control p<0.05.

Pentobarbitone sodium (35 mg/kg) and barbital sodium (200 mg/kg) were administered i.p. 30 min after pretreatment of extract or chlorpromazine HCl (1 mg/kg i.m.) to various groups of mice to evaluate the effect of the extract on barbiturate sleeping time[7]. The sleeping time was measured as a time interval between loss and regain of righting reflex. Similar experiment was also performed in mice treated chronically for 5 d with pentobarbitone sodium (35 mg/kg i.p.) only to stimulate the liver microsomal enzyme system and then on next day pentobarbitone sleeping time was studied as described. The extract treated group chlorpromazine 1 mg/kg pretreatment enhanced the pentobarbitone sleeping time in single as well as in chronically treated mice significantly. This pre-treatment also potentiated barbitone sodium sleeping time as shown in Table 2.

**TABLE 2:** EFFECT OF ETHANOL EXTRACT OF SEED OF *STERCULIA GUTTATA* ON BARBITURATE SLEEPING TIME

Pre-treatment	Pentobarbitone sodium sleeping time (min, mean±SEM)		Barbitone sodium sleeping time (min, mean±SEM)
	
	Acute	Chronic#	
Control	22.50±1.31	9.83±0.75	105.0±1.31
Extract (500 mg/kg)	33.50±.16*	8.17±1.02	133.5±3.61*
Extract (1000 mg/kg)	53.50±8.82*	15.67±2.57	159.5±8.82*
Chlorpomazine HCl (1 mg/kg)	54.67±1.31*	20. 67±1.88*	174.16±3.16*

n=6, All values of sleeping time are expressed as mean±SEM,

* Significant as compared to control p<0.05,

# Pretreatment of single dose of pentoberbitone sodium 35 mg/kg I. P. (5 d).

Statistical analysis of results was carried out to calculate mean±SEM. Further analysis was carried out by student's t-test to calculate significance of results. P values less than 0.05 were considered as significant.

Oral administration of *S. guttata* seed ethanol extract at 2000 mg/kg showed no toxicity up to 14 d in Swiss mice. The extract showed reduction in exploratory activity in the hole-board method, which is in support of the observation made in general behavior studies. However, this reduction is less as compared to chlorpromazine treatment group. The extract also potentiated the pentobarbitone- and barbital-induced hypnosis in normal mice, in single dose treatment as well as chronic pentobarbitone treatment group. It is well known that chronic treatment of barbiturates are reported to induce microsomal enzymes, leading to fast metabolism, so also there is development of neuronal tolerance[8], thereby reduction in hypnotic activity. Barbital sodium is known to have CNS depressant activity and is not degraded by liver microsomal enzyme system. Thus, there is significant potentiation of hypnotic activity of pentobarbitone, barbital sodium and chronic pentobarbitone treatment due to extract pretreatment. Usually CNS depressant drugs are known to act through GABA receptors[9] leading to reduction in exploratory activity[10] as well as potentiation of barbiturate sleeping time. In the present studies similar results were recorded after pre treatment of extract of *S. guttata* seeds, confirming the folk medical practice or claim.
